# Sexual violence along migration routes: A qualitative systematic review and evidence synthesis

**DOI:** 10.1371/journal.pone.0355474

**Published:** 2026-08-13

**Authors:** Alexandria Innes, Merili Pullerits

**Affiliations:** 1 International Politics, School of Policy and Global Affairs, City St George’s, University of London. Northampton Square, London, United Kingdom; 2 Violence and Society Centre, City St George’s, University of London. Northampton Square, London, United Kingdom; Omeo Kumar Das Institute of Social Change and Development, INDIA

## Abstract

**Background:**

This systematic review and evidence synthesis sought to systematise and synthesize qualitative evidence of sexual violence experienced by migrants travelling without state authorization, during the course of their journeys.

**Methods:**

Following a Population-Exposure-Outcome research design, where the population was migrants in transit, the exposure was a journey across state borders without state authorisation, and the outcome was sexual violence, we followed the Cochrane guidance for systematic review. Searches produced 49 studies that mentioned sexual violence along irregular migration routes, with 27 containing sufficient data for full inclusion and analysis. We applied a thematic coding schema across six descriptive codes.

**Results:**

This systematic review finds that sexual violence is widespread in the context of migration without state authorisation, and structurally embedded feature of undocumented migration. Findings were grouped around the six themes of agency, economics, climate of violence, normalisation of violence, perpetrators, and gender. Data showed that migrant women expressed agency by adopting preventative or protective measures against expected sexual violence, at times including engagement in unwanted sexual contact. Economic deprivation intersected with sexual violence during migration journeys, increasing risk of sexual violence. The most severe cases of sexual violence were not mitigated by money. In some cases sexual violence victimisation was considered to be the only available way to prevent deportation or death. Sexual violence was severe and indiscriminate, and included forced witnessing, and forced sex act perpetration on other migrants. Sexual violence along migration routes is executed by various types of perpetrators, including organised and systematic perpetration by state agents such as border guards and police, and by smuggling and trafficking gangs. Sexual violence is also carried out by opportunists who are often migrants in transit, or people who reside along the route. Sexual violence is used against men, women, trans and non-binary migrants, and people of all ages, but there are important gendered differences in the experience of sexual violence before and during a migration journey.

**Conclusions:**

Sexual violence along irregular migration routes is an urgent issue demanding a response. Sexual violence is structurally embedded in the governance of international migration, suggesting the need for significant systemic change. This problem requires dedicated attention and resources directed towards prevention and protection.

## Introduction

While all types of violence happen in the context of migration without state authorization, states have no directly tangible and clearly articulated incentives to protect migrants who travel in this way from violence. Indeed, all types of violence against migrants are evident in data on migrant journeys [1–3]. Migrants in transit, particularly those who travel informally without documents, are exceptionally vulnerable to exploitation from criminal gangs and smuggling networks [1], abuse by border forces, corrupt policing [4], and exposure to petty crime and violence during the journey [1,5,6].

The systems and structures that govern international migration without state authorisation create conditions of vulnerability for migrants, and sexual violence requires separate attention. Such systems and structures include formal immigration governance, which restricts so-called ‘legal’ routes, and therefore gives rise to migration without state authorisation [7,8]. They also include the infrastructure of immigration control (policing, border guards, coastguard patrols, checkpoints), which must be evaded by those on irregular or clandestine routes. The systems of structures that govern movement without state authorisation also include non-state actors such as NGOs and charitable organisations that afford temporary support and shelter [9,10], and criminalised networks that facilitate illicit border crossing [11]. Together these various agents, actors, institutions and organisations form the infrastructure of international migration without state authorisation both formally and informally. We refer to this generally as the ‘immigration system.’

Moreover, sexual violence in any context has unique causes and consequences [12,13]. In cases of sexual violence victimisation, reporting follows distinct patterns, linked to different social meanings and gender dynamics [14,15]. The legal and policy responses are often necessarily distinct from other types of violence, because of how it sits in the context of consent, coercion and more general culture [16,17]. Thus, in general there is good reason for treating sexual violence differently from other more general forms of violence.

## Study rationale and objectives

Despite barriers to disclosure (1), there is evidence of sexual violence along known routes where people migrate without state authorization [5,18–20]. Border policing means that forced migrants who cross international borders often travel with help from criminal gangs and smuggling networks, which produces risks of entrapment in a form of indentured sexual violence [18]. Migrants who travel to destinations outside of their home country to fulfil employment contracts for low-skilled labour are also particularly vulnerable, often finding that promised work is different from expectations, employers are abusive, and even that prostitution is the only option to pay debts incurred in transit [21]. Violent bordering policies make it impossible for at-risk migrants to report violence to authorities [3]. Fear of removal (often to unsafe locations) deters at-risk migrants from seeking support and protection from specialist services [3,22].

While important critiques of state governance and control of the international migration system have been launched, particularly with regard to the escalating number of migrant deaths in the Mediterranean and in the Mesoamerican corridor [23–26], there has been little change in violence against migrants, migrant injury and death over the last decade [27,28]. Indeed, states have tended to adopt the principle of deterrence to combat unauthorized migration, yet deterrent measures can exacerbate the conditions that produce violence and death where migrants most lack recourse to protection [23,25,29].

Drawing from theories of state sovereignty, security and citizenship suggests there is little or limited incentive for states to prevent sexual violence against migrants who might be travelling without authorization or be in the process of escaping adversity in their home state [23,30,31]. These individuals who are not part of citizenry cannot easily call upon available mechanisms to report and seek support and retribution for crimes of sexual violence perpetrated against them. Yet, there is precedent to consider sexual violence an international ‘wrong’ and to advocate for the consolidation of action against it (for example in United Nations Security Council Resolution (UNSCR) 1825, which established sexual violence in conflict as warranting consolidated international action).

Some existing research has examined violence associated with insecure migration status. A 2022 study found a high prevalence of gender-based violence amongst migrants and especially amongst vulnerable groups including undocumented migrants [32], and specifically called for further study of experiences of gender-based violence during migrant journeys. Our study responds to this gap in the context of sexual violence specifically. We extend existing knowledge through a qualitative systematic synthesis focused on sexual violence during unauthorized migration journeys. A recent systematic review [3] found that sexual violence was the most commonly referenced form of violence linked to insecure migration status, and this violence was closely associated with being a migrant in transit without state authorization and avoiding inspection by the state, as well as with lacking immigration status in a given destination country. However, that systematic review limited the study of sexual violence to direct physical violence so did not analyse broader forms of sexual violence such as sexual coercion or transactional sex. It also noted the need for more in-depth research in this area. Beyond this, studies adopting a wider conceptualization of sexual violence along undocumented migration routes have largely been confined to specific geographic contexts, focused for example only on the Central American corridor, North Africa, and the Mediterranean Sea route [33–35]. To date, there has been no comprehensive analysis that brings this dispersed evidence together to map where sexual violence that happens specifically during migration journeys is documented, under what conditions, and how it is interpreted across different political and geographic settings.

This current study addresses these gaps by synthesizing global qualitative evidence on sexual violence experienced during unauthorized migration journeys. Following the PRISMA guidelines for systematic review and meta-analysis [36,37] and using a thematic synthesis approach, we provide the first systematic qualitative review that explicitly links sexual violence to undocumented migration journeys. Our review maps the routes and geopolitical contexts where sexual violence is documented, identifies the circumstances and perpetrator types involves, and asks how sexual violence is framed and explained across studies. In doing so, we not only highlight where sexual violence is acknowledged globally, but also reflect on how research trends focus on certain regions while leaving evidence gaps elsewhere [38].

## Methodology

We conducted a thematic synthesis [39] of qualitative studies that reported experiences of sexual violence that happened during the course of a migration journey without state authorization. We follow the Cochrane guidance [40] for undertaking a systematic review and the Preferred Reporting Items of Systematic Reviews and Meta-Analysis (PRISMA) reporting checklist [36]. The protocol was registered on the PROSPERO database in January 2025 (reference CRD42025637870).

### Eligibility criteria

We included primary qualitative studies (and mixed methods studies that include a qualitative component) that document experiences of sexual violence against people in transit, while travelling without authorization. We excluded work that relied only on conceptual development or that only provided quantitative with no qualitative data. We included both peer reviewed academic research and grey literature in the form of doctoral theses and non-governmental organisation reports, as the inclusion of grey literature helps minimise publication bias and ensure a more comprehensive understanding of the available evidence [41]. Additional sources such as book reviews, conference papers and editorials were excluded. We included studies published since 1 January 2010, reporting violence that occurred since 2000, and no restrictions were placed on study setting (country) and source language. No results were returned outside of the language competency of the researchers.

As recommended for qualitative reviews, we followed a Population-Exposure-Outcome (PEO) design. The population were migrants in insecure status, following Innes’ definition [42]. The exposure was an undocumented and unauthorized migration journey, which was defined as being in transit, already outside of the home country, with no status requested or acquired in a destination country at the time of violence occurring. This meant that violence was always a function of the journey. Cases of sexual violence in a destination country, such as intimate partner or family violence following immigration, and cases where the purpose of migration was sexual exploitation in a destination country were not included unless the sexual violence happened during and as a consequence of the journey.

The outcome was sexual violence, which followed the World Health Organization’s definition of sexual violence. In line with this definition, we included verbal, physical, and emotional forms of sexual violence, and threat of violence [43].

### Search strategy

The search strategy combined three concept clusters: sexual violence, migration and methods. The concept clusters were developed by the first and second reviewers, with opinion sought from experts in violence and migration studies external to the reviewers. A Boolean search was carried out to comprehensively gather articles citing the separate clusters (OR search) and then combine the clusters with each other (AND search). See S2 Table for the full search strategy.

Database selection was based on previous reviews of violence related to insecure migration status that combined areas of expertise across authorship [2,3]. The five selected databases were *Embase*, *Social Policy and Practice*, *Political Science Complete*, *Sociology Source Ultimate*, and *Web of Science Social Sciences Citation Index* and searches were run on 16 January 2025. These database searches were supplemented with searches of grey literature. Grey literature searches were conducted between March and April 2025 using relevant key words in the search function and/or hand searches of research or publication sections of websites of the following organisations: European Commission; Freedom Fund; International Migration, Integration and Social Cohesion Research Network; International Labour Organisation; International Organisation for Migration; La Strada International; International Federation of Red Cross and Red Crescent Societies; Derechos Humanos Fray Matias de Cordova; World Health Organisation; United Nations Office on Drugs and Crime; Walk Free Foundation; SEREDA Project. In addition, the PROQUEST database was used to identify potentially relevant theses and dissertations. All included studies were also subject to forwards and backwards citation tracking to identify potential additional studies for inclusion. Forwards citation tracking was conducted using *Google Scholar*.

### Study selection and data extraction

**Fig 1:**
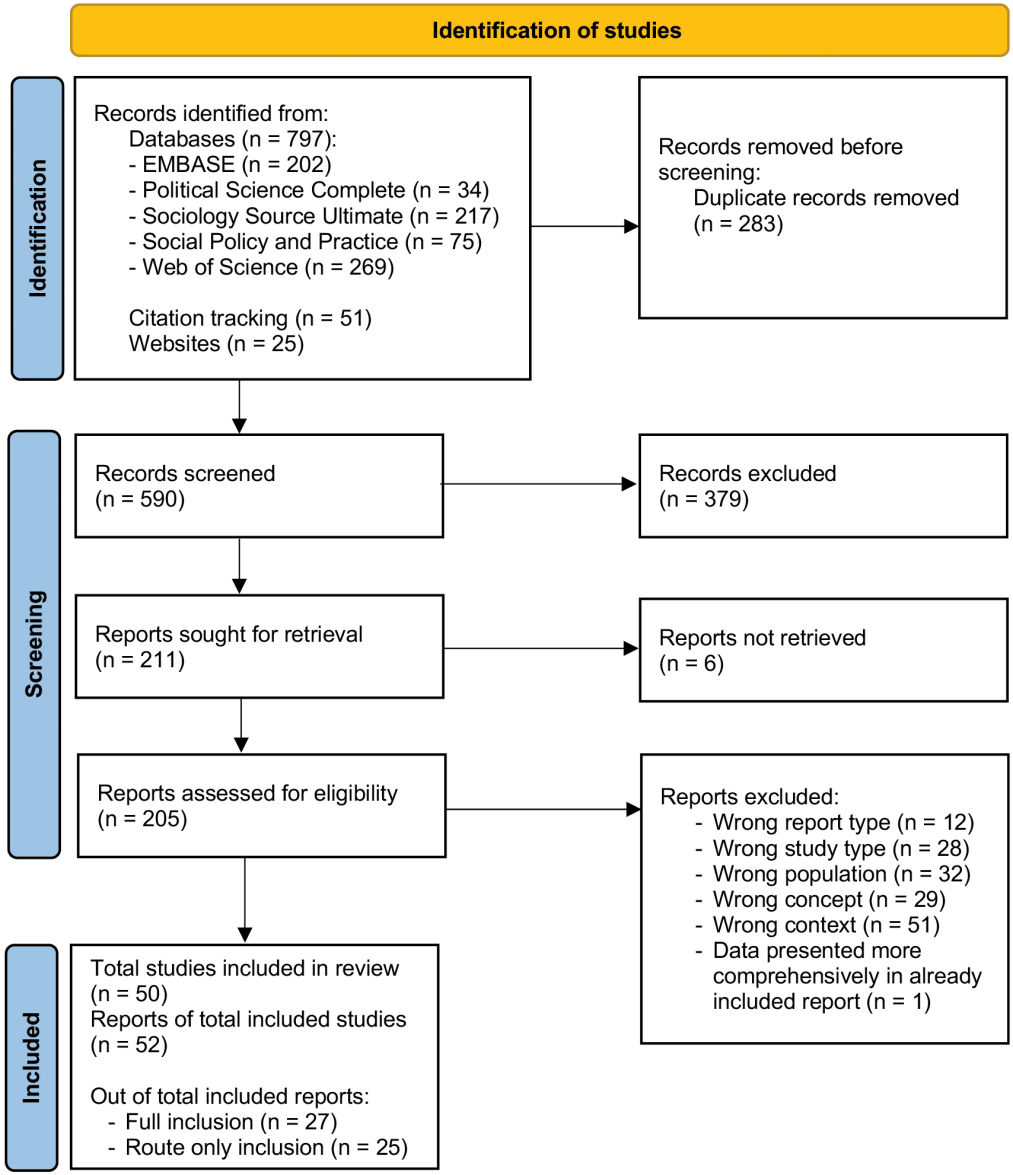
PRISMA Flow Diagram.

Zotero open-source referencing software [44] was used to deduplicate the search results, and the systematic review software Rayyan [45] was used for both title and abstract screening and for full-text screening. The first reviewer screened all titles and abstracts against the predetermined inclusion and exclusion criteria and studies that met the inclusion criteria at this stage proceeded to full-text assessment. Out of the 590 records initially screened at title and abstract stage, 211 proceeded to full-text screening, with 205 ultimately screened against the inclusion and exclusion criteria (see S3 Table) at full-text stage (full-texts of 6 records were not retrieved). The second reviewer independently screened 20% of records at both review stages and any disagreements were resolved through discussion until a consensus was reached. Following full-text screening, a total of 52 reports (representing 49 separate studies) met the inclusion criteria and were included in the review (Fig 1). These reports were categorised into two groups: 1) a full-inclusion group subjected to qualitative coding (n = 27) comprising studies with substantial qualitative extracts on sexual violence (3 + mentions), and 2) a route-only group (n = 25) made up of studies that met inclusion criteria but contained limited qualitative extracts on sexual violence (<3 mentions). While the full inclusion group is the main focus of the findings, the route-only group provides geographic context regarding research coverage of sexual violence along migration routes.

### Data collection process

A standardised Excel data extraction template was developed to capture relevant information from included studies. The template recorded the following categories: report ID and year, sexual violence type, source of violence information (e.g., first-hand account, witness etc.), country or region of violence, data collection method, participant characteristics, inclusion status (full inclusion or route-only), and notes. Data collection was divided equally between the two reviewers, and all data collection was checked by both reviewers.

### Quality assessment

For each study included on a full-inclusion basis, we carried out a risk assessment of bias using the Critical Appraisal Skills Programme (CASP) Checklist for Qualitative Research, which provides an assessment of a study’s validity, reliability, and applicability [46]. The following ten domains of the studies were assessed for quality: 1) Was there a clear statement of the aims of the research? 2) Is a qualitative methodology appropriate? 3) Was the research design appropriate to address the aims of the research? 4) Was the recruitment strategy appropriate to the aims of the research? 5) Was the data collected in a way that addressed the research issue? 6) Has the relationship between researcher and participants been considered? 7) Have ethical issues been taken into consideration? 8) Was the data analysis sufficiently rigorous? 9) Is there a clear statement of findings? 10) Is the research valuable? Quality assessment was conducted independently by two reviewers, and any disagreements were recorded, discussed, and resolved. Across studies, the main quality concerns related to limited reporting of researcher-participant reflexivity and ethical considerations, and in some cases, limited descriptions of analytical approaches. The full assessment for each of study included at full-text stage is reported in S4 Table.

#### Synthesis.

We adopted a thematic synthesis as the most suitable form of analysis for the research question, following Thomas and Harden [39] who recognise both the benefit of synthesizing qualitative research to inform policy and practice, but also the risk of falsely equivalating, or drawing comparison across, what is often deeply contextualised information.

All included articles were collected in a shared folder and initially subject to close reading by both reviewers. Data extraction from qualitative texts can be challenging given that raw data are rarely present in their entirety. Rather, data such as interview excerpts are often used illustratively to evidence the analysis, and at times first-hand accounts are described as ‘findings’ rather than ‘data’, despite limits to generalisability [39]. To ensure robust results, after close reading we only included texts that offered a minimum of three raw data excerpts detailing sexual violence, with the remaining included in the route-only group. This ensured that sexual violence was central to the study rather than mentioned tangentially. We applied the thematic codes to larger sections of the study: generally these sections were labelled as analysis or discussion sections. However, qualitative studies can be varied in their reporting style [39,47] and we included studies from a range of academic disciplines which are subject to different reporting norms. With this in mind, we used close reading to best identify the relevant sections of the text that offered qualitative evidence, discussion, and analysis of sexual violence along migration routes and focused on these sections for data extraction.

Thematic coding was carried out over three hierarchical levels. Level 1 coding was developed inductively from the close reading of texts and generated six descriptive themes across the included articles. A second close reading of the articles identified Level 2 interpretive themes which emerged from each descriptive theme, synthesizing the descriptive themes to draw out interpretations of the extracted data by enabling the translation of concepts across studies that might have used different language or various examples to detail similar phenomena. This also permitted us to compare and contrast *framing* of phenomena across studies. For example, in some studies, women pairing up with male travelling partners, even if this involved transactional sexual contact, was framed as a positive expression of agency, while in other studies the same activity was framed as evidence of victimization. In this way, synthesizing the descriptive codes across themes allowed us to categorise the nuance. Included articles were then double coded by the reviewers line-by-line for the interpretive themes. After reflection on the data and discussion, a third round of double-coding by both reviewers introduced Level 3 analytical themes for three of the six descriptive codes to better explore and deconstruct the translation and framing of concepts and phenomena across studies.

While this subsequent account of findings of the review includes some selected excerpts from the data, we have hesitated to recount the most brutal examples of violence provided in the evidence [34,48]. This was a reflective decision taken by the reviewers due to the potentially triggering nature of the violence described, including graphic description of sexual violence against children. The findings include excerpts detailing extreme violence, but are limited to those necessary to evidence the key themes.

## Findings

### Characteristics of Included studies

This systematic review included 27 studies that described or analysed detailed qualitative evidence of sexual violence experienced by migrants during the course of their unauthorised migration journey [5,18,19,33,34,48–68] with full details included in Table 1, and 25 studies that referenced sexual violence although did not include sufficient detail or discussion of the nature of the violence for full inclusion [35,51,69–90] with full details included in Table 2. Nevertheless, this provided insight into the migration routes most commonly studied where sexual violence is a known problem.

**Table 1:** Characteristics of Studies, full inclusion.

Author(s) and year	Region of violence	Data/method	Population	Sexual violence type	Source of violence data^1^
Adeyinka et al. 2023	Niger-Libya	Semi-structured interviews and questionnaires	31 female Nigerian trafficking victims, aged 16–35	Coercion, physical force	First-hand and witness accounts
Brigden 2018	El Salvador through Mexico to the US	Semi-structured interviews and participant observation	90 migrants from El Salvador, just under half women	Physical force	First-hand and witness accounts
Cenat et al. 2020	Along ‘The Road’ (Brazil and Costa Rica)	Semi-structured interviews	16 Haitian adults (6 women, 10 men), aged 22–58	Physical force	First-hand and witness accounts
Chynoweth et al. 2022	Multiple regions: Myanmar-Bangladesh; central Mediterranean migration route through Libya; and East-Central Africa	Semi-structured interviews and focus groups	310 refugees aged 15–65 in 55 separate focus groups (for boys, men, women, men with disabilities, people with diverse SOGIESC) across three settings; and 148 key informants	Coercion, harassment, physical force,	First-hand, witness and key informant accounts
Cook Heffron 2019	Central America-Mexico-USA	In-depth interviews	19 adult women migrants from El Salvador, Guatemala, and Honduras, average age 35	Coercion, physical force	First-hand accounts
Cortés 2018	Central America-Mexico-USA	In-depth interviews	30 Central American migrants and activists/stakeholders	Coercion, physical force	First-hand accounts
Elmagboul et al. 2017	Ethiopia-Sudan	In-depth interviews	15 female Ethiopian irregular migrants, aged 15–29	Harassment, physical force	First-hand and witness accounts
Gebreyesus et al. 2019	Northeast Africa to Israel	In-depth interviews and focus groups	12 Eritrean refugees (6 women, 6 men) in interviews and 44 Eritrean focus group participants in 8 separate focus groups (4 with women, 4 with men)	Physical force	First-hand and witness accounts
Ghebrezghiabher and Motzafi-Haller 2015	Northeast Africa to Israel	In-depth interviews and reviews of documents/radio interviews	10 Eritrean women	Physical force	First-hand and witness accounts
Goodson et al. 2020*	Europe/North Africa	Semi-structured interviews	36 refugees and migrants (31 female, 5 male) from Sub-Saharan Africa	Coercion, physical force	First-hand accounts
Infante et al. 2013	Mexico	In-depth interviews	22 Central American migrants (8 female, 14 male); and 10 key informants	Against minors, harassment, physical force, transactional	First-hand and key informant accounts
Infante et al. 2020**	Mexico and Central America	Semi-structured interviews	58 Central American migrants (28 women, 30 men), mean age 30.7	Coercion, physical force, transactional	First-hand and witness accounts
Izcara Palacios and Andrade Rubio 2022	Mexico and Central America	In-depth interviews	24 Central American migrants (15 women, 9 men), aged 19–26	Coercion, harassment, physical force, transactional	First-hand accounts
Keygnaert et al. 2014	In and around Morocco	In-depth interviews	154 Sub-Saharan migrants (60 female, 94 male), mostly from DRC and Cameroon, and mostly aged 19–29 years	Coercion, harassment, physical force, transactional	First-hand and witness accounts
KIND and CDH Fray Matías 2017	Mexico and Central America	Interviews and analysis of case files	96 Central American migrant children (49 girls, 47 boys), aged 12–17; and 78 stakeholders	Against minors, coercion, physical force, transactional	First-hand, witness and key informant accounts
Letona et al. 2023	Mexico and Central America	In-depth interviews	39 Central American migrant women and girls, aged 15–46	Physical force, transactional	First-hand and witness accounts
Leyva-Flores et al. 2019**	Mexico	In-depth interviews	58 Central American migrants (28 women, 30 men), mean age 30.7	Physical force, transactional	First-hand accounts
López-Domene et al. 2019	Sub-Saharan Africa/Mediterranean Sea	In-depth interviews	10 African migrant women, aged 19–39; and 10 key informants	Coercion, physical force	Witness and key informant accounts
Matose et al. 2022	Botswana-Zimbabwe border	Semi-structured interviews	15 female irregular migrants, aged 17–50; and 4 key informants	Physical force	First-hand, witness and key informant accounts
Obach et al. 2022	South America	In-depth interviews	17 Latin American migrants, aged 18–24; and 18 key informants/stakeholders	Coercion, physical force, transactional	First-hand, witness and key informant accounts
Pertek et al. 2021*	MENA region and Sub-Saharan Africa	Semi-structured interviews	68 refugee victims of sexual and gender-based violence (13 men, 54 women, 1 other) from the MENA region and Sub-Saharan Africa; and 26 service providers	Coercion, harassment, physical force, transactional	First-hand and witness accounts
Preko 2022	North Africa	Interviews and analysis of news articles	5 West African undocumented migrants (4 male, 1 female)	Coercion, physical force	First-hand and witness accounts
Rubini et al. 2024	En route between West Africa and Italy	Retrospective qualitative analysis of clinical interview transcripts (sexual violence service)	43 Sub-Saharan African women, aged 19–48	Physical force	First-hand and witness accounts
Soria-Escalante et al. 2022	Mexico	In-depth semi-structured interviews	10 Latin American migrant adult women	Coercion, physical force, transactional	First-hand accounts
UNICEF 2016	Various, en route to Northern France transit zone	Individual and group semi-structured interviews	61 unaccompanied migrant children, aged 11–17	Against minors, coercion, physical force, transactional	First-hand and witness accounts
Vogt 2012	Mexico	Interviews (including oral histories) and ethnographic observations	~50 Central American migrants and shelter staff/stakeholders (limited demographic details)	Coercion, harassment, physical force	First-hand, witness and key informant accounts
WRC 2019	Central Mediterranean route	In-depth semi-structured interviews and focus groups	52 male refugees, aged 15–40	Physical force	First and witness accounts

1 Witness includes direct witnesses and indirect migrant witnesses, who are those reporting what they were told by others. It excludes key informants reporting what migrants told them, which are labelled separately as ‘key informant accounts’.

*The papers are part of the same wider study.

**The papers are part of the same wider study.

**Table 2:** Characteristics of Studies, route only.

Author(s) and year	Region of violence	Data/method	Population	Sexual violence type	Source of violence data^1^
Barbuzano et al. 2020	Morocco-Spain	Creative biographical narrative interviews	13 Nigerian women, aged 18–34	Coercion, physical force, transactional	First-hand accounts
Chentoufi and El Allame 2025	North Africa	Semi-structured interviews	15 undocumented Sub-Saharan migrants (5 women, 10 men), aged 16–42	Coercion, physical force	First-hand and witness accounts
Chynoweth et al. 2020*	Multiple regions: Myanmar-Bangladesh; central Mediterranean migration route through Libya; and East-Central Africa	Semi-structured interviews and focus groups	310 refugees aged 15–65 in 55 separate focus groups (for boys, men, women, men with disabilities, people with diverse SOGIESC) across three settings; and 148 key informants	Coercion, physical force	First-hand and key informant accounts
Cueva-Luna and Terrón-Caro 2014	Mexico	In-depth interviews	26 migrant women from Mexico, Central America and South America, transiting Mexico’s northeastern border	Coercion, harassment, physical force	First-hand and witness accounts
Erazo Caravantes et al. 2019	Mexico and Guatemala	Focus groups, in-depth interviews, and a ‘legislative theatre’	Interview population: 48 Central American migrant women; and 42 stakeholders Population for the 24 focus groups: total of 230 women and 43 men from Mexico, Guatemala, El Salvador and Honduras	Coercion, harassment, physical force,	First-hand and key informant accounts
Freedman 2016	Various, en route to the EU	Depth interviews	60 refugees (40 female, 20 male) from Syria, Afghanistan, Iran and Eritrea; and unknown number of key informants	Coercion, harassment, physical force, transactional	First-hand and key informant accounts
Goodson et al. 2021	On journey ‘hotspots’ from MENA to UK	Semi-structured interviews	22 migrants (15 female, 6 male, 1 transgender) from the MENA region, majority in their 30s	Transactional	First-hand and witness accounts
Hlatshwayo 2019	Zimbabwe-South Africa	In-depth interviews and documentary analysis	35 Zimbabwean women, aged 20–45	Harassment, physical force,	Witness accounts
Infante et al. 2012	Central America and Mexico	In-depth interviews	22 Mexican and Central America migrants (4 female, 18 male)	Physical force	Witness accounts
IOM 2024	Southern Route (Africa)	In-depth interviews	60 migrants (10 female, 50 male) from Ethiopia, Somalia and Eritrea, aged 20–35; and 46 key informants	Coercion, harassment, physical force	First-hand, witness and stakeholder accounts
Jimenez-Lasserrotte et al. 2020	Mediterranean route	In-depth interviews	26 adult irregular female migrants mainly from West Africa, who arrived in Spain by small boat	Coercion	First-hand accounts
Khan and Caihol 2020	Turkey (en route from Pakistan to France)	In-depth interviews, a focus group and observations	13 Pakistani migrant men, aged 23–56	Coercion, physical force	First-hand and witness accounts
Kiss et al. 2022	Uganda and Nigeria	Semi-structured interviews	16 female adolescent victims of trafficking from Rwanda, Uganda and Nigeria, who were under 18 when they migrated	Against minors, coercion, physical force	First-hand and witness accounts
Lilja et al. 2020	En route to Europe, with Libya mentioned explicitly	Analysis of qualitative reflective journals kept by counsellors from NGOs supporting migrant women	Women migrant victims of gender-based violence	Coercion, harassment, physical force	Key informant accounts
Linthout et al 2025	En route to Europe, with Libya mentioned explicitly	In-depth interviews and participant observation	39 migrant men from mainly East Africa and MENA, aged 16–45 years	Coercion, harassment, physical force,	First-hand and witness accounts
Pertek 2022	North Africa (primarily Libya)	In-depth interviews	15 female sub-Saharan refugees and refused asylum seekers, aged 18–44	Physical force	First-hand accounts
Pertek and Phillimore 2022	Various, but many routes into Europe from Sub-Saharan Africa and MENA	Semi-structured interviews	166 forced migrant victim-survivors of violence (131 women, 35 men) from MENA and Sub-Saharan Africa, with majority in their 20s and 30s; and 107 service providers	Coercion, physical force, transactional	Witness and key informant accounts
Ramage et al. 2023	Central America and Mexico	Semi-structured interviews	30 asylum-seeking women from Mexico and Central America, aged 18–41	Coercion, physical force	First-hand accounts
Sarkin and Morais 2024	Northeast Africa to Israel	Depth interviews	11 refugee and asylum-seeking Eritrean women; and 12 key informants	Harassment, physical force, transactional	Witness and key informant accounts
Servan-Mori et al. 2014	Mexico	Depth interviews	35 Central American migrants (28 men, 7 women)	Coercion, physical force	First-hand accounts
Sladkova 2016	Central America and Mexico	In-depth semi-structured interviews	20 undocumented Honduran migrants (6 female, 14 male)	Against minors, physical force	Witness accounts
Topak 2024	Sahara	Interviews	24 male migrants from Eritrea, Somalia and Sudan, mostly between aged of 20 and 30	Physical force	Witness accounts
Velasco and Heredia 2023	Central America and Mexico	Narrative testimonies	9 women migrants from Honduras and El Salvador, aged 18–38	Harassment, physical force	First-hand accounts
Willers 2016	Mexico	Interviews	34 migrants (31 women, 3 men) from Honduras, El Salvador and Guatemala, aged 19–56	Coercion, physical force, transactional	First-hand accounts
Zaman et al. 2024	South America	Secondary thematic analysis of 265 ‘micronarratives’ gathered using a sensemaking approach	265 female Venezuelan migrants/refugees, mean age 25.8	Coercion, harassment	First-hand accounts

1 Witness includes direct witnesses and indirect migrant witnesses, who are those reporting what they were told by others. It excludes key informants reporting what migrants told them, which are labelled separately as ‘key informant accounts’.

*The papers are part of the same wider study – this paper is part of the same wider study as Chynoweth et al. 2022 in the full-inclusion.

The reports categorised for full inclusion reported qualitative data from a total of 1,221 migrants and 294 key stakeholders and service providers, plus an additional approximately 100 participants made up of both migrants and stakeholders, where precise counts were not provided.

Across all 52 reports, the most commonly studied routes fell into two broad categories. One category involved the route north from Central America through Mexico towards the USA (n = 18). Most documented instances of violence along this route took place in Mexico during transit. The second category involved routes across Africa towards Europe (n = 12), which featured prominently in the fully included detailed qualitative studies. These included routes from both West Africa (Nigeria-Niger) and East Africa (Horn of Africa-Sudan), transiting through Libya towards Europe. Across these African routes towards the EU, violence was described as saturating the journey, with a particular concentration of evidence relating to violence in Libya. Other routes that were studied included a route across the South of Africa (n = 2), or central and sub-Saharan Africa (n = 7), a South American route through Venezuela towards Chile (n = 2), and between Costa Rica and Brazil (n-1) and a North-East African route though Egypt (n = 2), a route that transited through the USA to Canada (in some cases with a long-term stop in the USA before moving northwards) (n = 1), a route through Turkey (n = 1), a route from Myanmar towards Bangladesh (n = 2), and a route through Calais towards the UK (n = 1). Some studies detailed multiple routes, others were not specific about the location of violence on the journey.

## Data synthesis: Descriptions and patterns of sexual violence along international migration routes

### Level 1 descriptive themes

Six descriptive themes were generated by the initial close readings of texts. These were as follows: 1. ‘Agency’, referring to the general appearance of references to migrant agency across the included texts whether these were positive or negative. 2. ‘Economics’, which referred to economic hardship and structural disadvantage that was linked to an outcome of sexual violence. 3. ‘Climate of violence’, which referred to all references to sexual violence that were linked to other forms of extreme violence, brutality and death. 4. ‘Perpetrators’, categorising the identity of the perpetrators detailed across the included studies. 5. ‘Normalisation of violence’ was noted in reference places where sexual violence was accepted as inevitable on a migration journey. And finally 6. ‘Gender’, referring to gender-based or gendered dynamics evident in sexual violence.

### Level 2 interpretive codes

The second level of coding was developed in a second round of close reading across the descriptive themes, and produced a synthesis of data extracted from the included texts that offered insight into how the descriptive themes translated and travelled across the studies. This allowed us to review patterns across the treatment of descriptive themes, as well as how the studies, routes, or characteristics of the studies differed across these themes. The interpretive codes are summarised in Table 3:

**Table 3:** Interpretive Code Summary.

Level 1 Descriptive Themes	Level 2 Nuance generated from synthesis
Agency	Transactional violence Responsibilisation
Economics	Entrapment Survival
Climate of violence	Visibility of death Spatial and temporal
Perpetrators	Organised and formal Direct interpersonal Structural/ systemic violence
Normalisation	Inevitability Widespread ≠ normalised
Gender	Characteristic differences by gender

### Agency

The descriptive theme of agency generated several insights. Across the included studies, two main framings of agency were identified. The first framing portrayed a lack of agency amongst migrants in transit, although this lack of agency was varied in terms of explicit use of violence. For example, as a migrant in one interview states ‘if we have to get things with sex, then we have to have sex’ [33]. This suggests a transactional element to sexual violence, and an acceptance of the condition of sexual violence, even though the violence is not physically forced. At times, migrants engaged in commercial sex work, but this decision was contextualised in destitution and a lack of alternatives during transit [49–51].

The portrayal of sexual violence as transactional was common [5,51,10,91]. All of these examples were coded as sexual violence for this review, because of their contexts, including clear articulation that sexual contact was unwanted, power imbalance such as in the context of immigration control or reliance on smugglers, threat of greater violence and death, and destitution or absolute economic need.

In other instances, the lack of agency was more directly linked to physical violence:

‘We were travelling as a group of men and women and came across a group of soldiers. They made us sit down and threatened to arrest and deport us. They started looking amongst us and picked the younger women, and raped them not so far from where the group was sitting. It was so painful, and there was nothing we could do’ [52] p.5.

Such examples demonstrate the lack of agency or the lack of alternative options available to migrants. Keygnaert et al [19] report that the impossibility to resist violence or escape from violence was emphasized by both victims and witnesses to violence interviewed in the study.

The second of the two framings of agency was its characterization as a theme that highlighted the methods migrants used to reclaim agency in the context of sexual violence. These included things like dressing ‘like a boy, to escape’ being picked out for abuse [18], p. 12, or agreeing to a ‘relationship’ with captors as a survival strategy. The excerpt in Adeyinka detailing this makes explicit that this was a strategy adopted as a result of violence, rather than a choice:

‘They think I want to f*** that wicked man [smuggler and trafficker] that tear my body with his big thing? I had no choice now. It was the only way to survive’ [18], p.12.

There were also differences across studies in terms of the *perception* of agency, which we coded as ‘responsibilisation.’ For example, male migrants often perceived women to possess an ability to negotiate because they had the capacity to ‘offer’ sex as a ‘ticket to travel’ [5,33,10]. Similarly, the acquisition of contraception before a journey might be positioned as consent, or the knowledge of sexual violence along the route produces a ‘blame the victim’ mentality [53]. Across the included studies, the various ways agency appears positions that agency as existing only within the limits imposed by significant structural constraints, forms of oppression, and interpersonal power dynamics.

### Economics

Economic need was often established as a push factor for migration in the studies, indicating that economic need is linked to irregular migration as a motivation or cause: those in the most extreme economic need have limited options [49]. Economic need was also associated with entrapment in sexual violence, for example:

‘I realised that no one will help me raise my siblings … they are my younger ones, who will take care of them, if not me? It was while I was hustling and looking for a way to make enough money that I met a woman who promised me a job in Libya as a stylist.’ [18], p. 6.

Entrapment might be enforced with violence, or denial of freedom. In some cases in the West African context, it was enforced with juju rituals in which women were taken to a shrine to swear an oath to repay an amount of money determined by the trafficker [18].

Economics were linked to transactional sexual violence, particularly in the context of ‘survival sex’ to meet the most basic of needs. For example, on the central American route an excerpt reports:

‘I had sex with someone, even for an order of tacos in Mexico. It happened to me on the way, too. I had to have sex with someone in exchange for a glass of water’ [49], p.3.

In this way the sexual violence that emerged linked to economic need was characterised by two dynamics. One was economic need leading to migration that produces migrants who travel the most dangerous routes, with or without engaging smugglers, but vulnerable to exploitation along the route. The second is economic need that leads to structurally or circumstantially coerced engagement in sex work or transactional sex during transit.

### Climate of violence

The studies outlined many examples of extreme violence and brutality. These were often connected with travel through West and North Africa [18,19,48,54], but were also present elsewhere, including the Central American route [5,55]. It was evident across these studies that death is visible along migrant transit routes, via the visibility of bodies, and witnessing killing. In this way, sexual violence happens in a context of being surrounded by death: threats of violence are reinforced by witnessing those who resist smugglers and traffickers being brutalised and killed or left for dead [34,48,65].

There is a climate of violence surrounding irregular migration routes that is tangible throughout the evidence in the included studies. This is *temporal* where violence pre-migration is the, or one of multiple, motivators for migration [65]. The temporal aspect is also linked to being on the journey: migrant journeys are not linear or constantly in motion [92], and evidence of the phenomenon not of *waiting* for an opportunity to move on, but more brutally of kidnapping and imprisonment are evident in some of the excerpts. For example, Chynoweth et al. [48] refer to men being held in containers, and Gebreyesus et al. [34] describe ‘torture camps’ where women can end up trapped:

‘Sarah was already a captive there. She was trapped there for a long time because she could not pay her ransom. She was raped and tortured for many months’ [34], p. 735.

The climate of violence is also *spatial* in the sense that the journeys traverse hostile terrain where travellers are entirely dependent on smugglers or traffickers for their survival [34,48,65]. For example, an excerpt describes:

‘[t]he traffickers started to rape us on our way to Sinai. Every time we stopped for the night, the truck drivers came to where we, the women, slept, they used torch lights to select the beautiful one amongst us. They took her to a nearby place, raped her, then returned her to our sleeping area’ [56], p. 584.

The hostile and isolated desert route meant that there was no available escape route without risking death.

### Perpetrators

While victims and survivors of sexual violence were not exclusively female, the perpetrators of sexual violence across the included studies were exclusively male. The most common perpetrators of sexual violence were the smugglers or traffickers that were transporting migrants, and border guards, police, and other officials [10], who were using their authority to coerce sexual contact. There were varying levels of systematization and various mechanisms of systematization apparent in the accounts. For example, an excerpt from Vogt [57] documents sexual assault at a militarised migration checkpoint:

‘Everyone was there, migration, the army, the Federal Police were there, there were so many of them. And they checked me, and as I was the woman who had come on the train, how they touched and groped me until I urinated on myself. I urinated because I was scared that they were going to rape me…’ [57] p. 212.

The presence of state officials including the police, collaborating in sexual assault, is suggestive that this is not rare: if the behaviour was unusual one would expect it to be hidden. Other examples of state-affiliated perpetrators include rape being used as a condition to pass through an immigration checkpoint [33,58], rape by police or the military while held in detention [18,54], or when in border areas [48,59]. Evidence also suggested collusion between police and criminal gangs, for example Ghebrezghiabher and Motzafi-Haller [56] include examples of police selling migrant women to traffickers. Rubini [54] reports that ‘individuals who paid for victims’ release from detention centres were the ones that sold them to brothels’ [54], p.7. Smugglers delivering migrants to, and entrapping migrants in prostitution was also evidenced [18].

There were multiple accounts of smugglers and traffickers using systematised sexual violence to control migrants. This included imprisonment, rape, sexual enslavement, forced nudity, sexual assault, forced prostitution, and forced perpetration of sex acts on other migrants [19,48,56].

There was evidence that perpetrators were often in a ‘protective’ role and then abused that position. These cases included police who are charged generally with public protection, but also paid smugglers who were hired to facilitate a migration journey [60,61] and also other migrants with whom female migrants entered a mutually beneficial friendship and were then coerced into sexual contact. For example:

‘He told me, “No, I only care for you as a daughter, and I love you as one, I want to help you because I know all you’ve done to come this far, like leaving your daughter behind, so I want to help your daughter and I will help you.” So, I came all the way up here, and when we got here, he became somebody else. He told me to pay him for all he had done for me, and all he had spent on me, so I told him “You know I don’t have any money,” so he abused me, he grabbed me by force…’ [62] p.1268.

There was some recognition in the data that travelling as a couple afforded benefits for both male and female migrants, although these relationships could also lead to different types of vulnerability. Perpetrators were also opportunists, including migrant men [34,49,57,62,63], and people along the route such as truck drivers, taxi drivers, and local citizens who lived along the common travel routes [19,50,52,56,58,64,69].

### Normalisation

The included reports provided evidence that sexual violence is known along the route to the extent that it is accepted as inevitable by travellers, understood to be likely and expected on the journey, cross cutting with the theme of agency, where women took measures before and during travel to prevent unwanted pregnancies. As evidenced in Letona et al [49]:

‘[The trip] scared me a lot. They gave me injections: what were [the injections] preventing? …At first, I didn’t ask myself why; why if they [parents] really knew that [rape] could happen to me, why were they sending their daughter there?” [49]p. 3.

As detailed by a migrant in another study ‘In general, the men smuggling us will be forcing girls to have sex at some point along the journey’ [34], p. 730. Infante et al [33] remark that ‘this perception of the inevitability of sexual violence and of the inability to control or prevent it, was prevalent in the discourse of study participants’ [33] p.1153. Sexual violence in many studies was discussed as part of the risk of undertaking a migration journey, expected, and while measures might be taken to mitigate the effects, could not be entirely avoided.

### Gender

Research on sexual violence implies an expectation of gendered victimisation, given the long history of violence against women, and an expectation that perpetrators of sexual violence are male. The cultural normalisation of sexual violence against female migrants raises themes of victim-blaming and responsibilisation [33,55]. Yet, perhaps the most significant finding in this regard intersects with the normalisation of sexual violence, which was (unsurprisingly) gendered female. Three reports focused on sexual violence against men [19,48,58], and it was clear in the data that such violence is widespread but not normalised. This is linked to the shame attached to disclosure of violence [48], but reluctant disclosure also leads to ongoing negative physical, mental, and social effects [19,48].

### Level 3 interpretive codes

The third level codes were applied only to three of the six descriptive codes: Agency, Climate of Violence, and Perpetrators. These were the codes with the greatest discrepancies in concept translation and framing across the studies, but these discrepancies formed some clear patterns indicating the need to break them down further. The ‘Agency’ code specified two dominant framings of agency across the included studies as detailed above. One tended towards a negative framing, where agency is deconstructed as a form of responsibilisation, or in more political terms, victim-blaming. In these cases, sexual violence was framed as ‘condoned’, with less attention paid to the structural constraints. The second framing of agency was positive where preventative or strategic action adopted by women in the context of (the threat of) sexual violence was framed as resistance. The third level coding recognised this distinction as a pattern whereby the framing of female agency was either positive [49,91] or negative [51,52] as it travelled across studies.

Climate of Violence also produced two sub-categories in the third level coding stage: one was to portray sexual violence as an additional tool used in a brutal toolkit of extreme violence. The other focused on the *protective* aspects of sexual violence, where the possibility of sexual violence was seen to reduce the likelihood of death. Again, the distinction was portrayed as a pattern across studies, where some studies highlighted the extent of violent brutality and sexual violence was integrated into this narrative [18,19,34]. Other studies focused on the perception of sexual violence as a constrained choice that offered protection, but was accepted because of its embeddedness in a violent migration journey [33].

**Fig 2:**
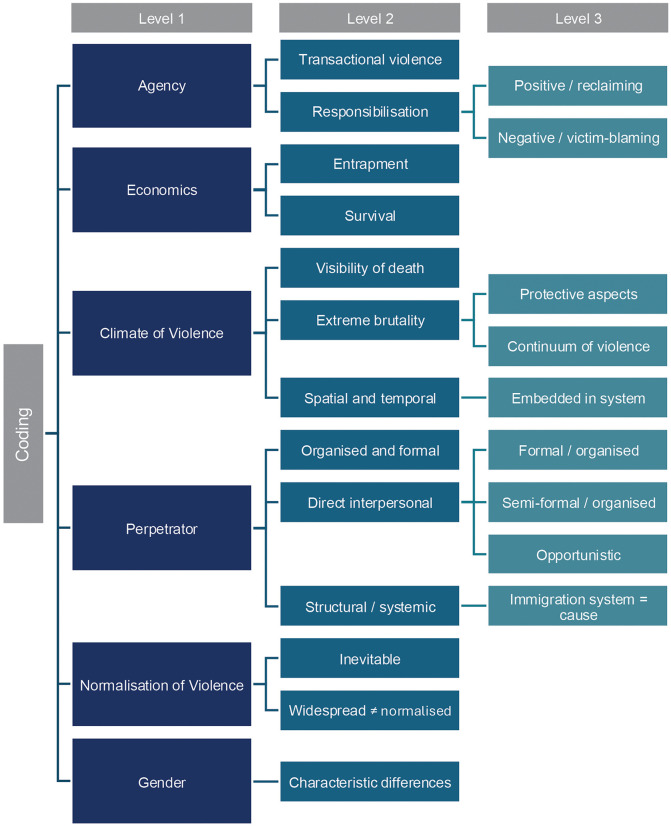
Hierarchical Coding Schema.

Finally, the theme of Perpetrators was divided into two sub-themes: Systematic violence referred to studies that acknowledge where dimensions of the system in which migration happens are permissive of sexual violence. In this case, the dynamics of the immigration system itself were constructed as a cause of violence [51,67,65]. Studies recognised the violence of individual perpetrators, but several also focused on immigration systems as a structural cause that highlights the systemic enablement of sexual violence [18,55,57,60,69]. The second sub-theme looked specifically at the perpetrator types at the individual level: for example types included formal (state, police, immigration officers, border control) and semi-formal such as smugglers and traffickers whose power is enabled solely by the immigration system [18,19,33,52,54,55,62] and then informal perpetrators, such as opportunists during the journey who may or may not be migrants themselves [49,57,62,63,66]. This framing focused on individual perpetrators and the relational power dynamics in specified situations of sexual violence. The full hierarchical coding schema can be found in Fig 2.

## Discussion

The following discussion traces analytic findings that emerge from systematic study of the routes, grouped around the six thematic codes detailed above, and recognising where the themes overlap.

### Agency: Transactional violence and responsibilisation

The first, and most dominant, framing of agency was to foreground the lack of agency on the part of migrant victims, and victim-survivors, of sexual violence. This intersected with other descriptive themes across the studies. For example, where economic need was most prevalent the routes travelled incurred the most danger of violence, including sexual violence. Additionally, the climate of violence and the extreme brutality documented across these studies further emphasised the lack of agency on the part of migrant victims and victim-survivors. Studies included examples of torture, of forced witnessing of sexual violence, of witnessing death that was a consequence of sexual violence, and of witnessing killing where violence was resisted.

However, a framing of agency asserted by migrants, whereby female migrants adopted birth control before travel to avoid unwanted pregnancy, and where migrants entered into partnerships with other migrants for mutual protection, was also present. In some of these cases sexual violence was constructed as transactional. Yet, the spectrum of transactional sexual violence included examples of sex acts to avoid destitution for needs as basic as food and water. It also included transactions to permit border crossing, and it included engagement in sex work. The acceptance of sexual violence to facilitate border crossing can be understood as constrained agency. The choices for migrants in this setting are limited even when there is no threat of killing or brutality. Condoning or accepting sex acts can be understood as a means of retaining a sense of power: where resistance might result in physical violence, acceptance can retain a sense of agency and dignity. As one woman explained: ‘The advantage of being a woman is that men will help you just to have sex with you. Just think of it as paying for protection with [your] body’ [5].

Geographically, the examples of transactional sexual violence tended towards the Central America – Mexico route, and in these cases men at times perceived women as having an advantage because they could use their bodies to pass through immigration checkpoints, or to appease border guards and police who might otherwise detain and deport them. In these examples, the migratory journey is the objective, and there is a general acceptance of the conditions of travel that are violent and at times brutal. In such cases the descriptive theme of agency intersects with the normalisation of violence on the journey: where the type of unwanted transactional sexual violence would be perceived as shocking in everyday life, on the migration journey it is construed as a means to an end [52]. This evidence is suggestive of the overall violence of the journey: sexual violence can be constructed as a lesser harm to suffer, with the risk of physical harm and death repeatedly demonstrated along the journey. The structural constraints on female migration are enhanced in this way. Women embark on migration journeys for multiple and complex reasons. The agency exercised in the act of irregular migration is subject to the constraint of sexual violence being likely while in transit. This can produce a victim-blaming logic for the lack of protection from sexual violence [10]. For example, women described as a ‘temptation’ for men, removing the agency behind violence from the perpetrator and shifting it to the victim [62]. For other women, sexual violence is posited as an advantage that lessens other physical risks along the route [33]. If sexual violence is positioned as a ‘cost’ of the journey in this way, attention is diverted from both the systemic conditions that permit it, and from the perpetrators,

### Economic entrapment and the means of survival

Economic need saturated the migration journey. In cases where poverty was a dominant push-factor for migration, the routes travelled tended to be more dangerous. Several accounts mentioned kidnapping by Bedouin traffickers who commonly used sexual violence and torture, and kidnapping was considered more likely if travelling without the help of a (paid) smuggler. Yet, paid smugglers only offered marginal protection against this type of kidnapping. For example, in one account a migrant described being instructed by a smuggler to bury herself in the sand to avoid kidnapping [34]. At times paid smugglers also sell migrants on to traffickers who then imprison and torture them [34,59]. While money can be a protective factor to ease migration, purchasing better guides, safer routes, and bribes for border crossing, money is not always effective protection.

In some accounts, economic entrapment occurred as a result of money ‘loaned’ for migration. It was relatively common on the West Africa – Libya route for passage to be arranged with the promise of expenses recouped, and the promise was ascertained via ‘juju rituals’ which targeted the family of the migrants as victims and used this threat to ensure compliance in long-term sexual violence during the journey and after reaching the destination country [18,67,93], although the latter was out of the scope of this review. In these cases the economic entrapment is constructed as a direct consequence of the necessary means of travel in order to cross the desert in Niger, and to cross borders without state authorization or the requisite travel documents. The debt incurred to arrange transit is illegal extortion, and entraps women in sexual violence in the form of forced sex work, for which all income services the debt.

Economic hardship was also common along the migration routes. Often, whether travelling with or without a smuggler, journeys can become longer than planned or expected, and money might run out. As detailed above, women report exchanging sex for basic survival needs such as food, water, and shelter [49]. There are also examples of sex being demanded as payment for protection and resources provided by fellow travellers or informal smugglers during a journey and after protection has been delivered [62].

In short, violence is a dimension of poverty [94], and poverty constitutes a risk factor for violence [95]. International irregular migration journeys are no exception. Where poverty is a significant factor in international border crossing, and where this border crossing happens through routes that are least costly, the risk of violence is greater. Yet, violence cannot be explained by poverty in these cases as the risk of violence is high along international migration routes when travelling without state authorization, both because there is little recourse for protection from violence, and because state actors are complicit in violence including sexual violence. Findings suggest that the unauthorised nature of the journeys and the consequent lack of recourse for protection from violence or accountability for perpetrating violence can explain violence on the journey, although poverty is an intersecting factor that increases risk of violence overall.

### Climate of violence and the visibility of death

The climate of violence along the route intersects with evidence that violence becomes normalised. However, the evidence for the interpretive theme focused most closely on types of spectacular violence, such as witnessing violent punishment including killing for refusing to comply with sexual violence, and forced witnessing of brutal sexual violence that resulted in death [19,34]. The proximity to violent death on migration journeys is described by participants across the studies.

Proximity to death is spatial in the sense that journeys transit through hostile terrain, often on foot. Travellers who cannot withstand the demands of the journey are often abandoned and left to die by smugglers and the rest of the group. Migrant interview participants describe viewing bodies as they travel through the desert, and the jungle, and seeing people fall from ‘La Bestia,’ the freight train through Mexico on which migrants typically ride atop the carriages [55,57,62]. The proximity to death is a reminder of the climate of violence in which the journey happens, suspending the normal ontological security of everyday life. The reality of hostile terrain reinforces the extent to which migrants rely on the smugglers who support their transit, hence a threat is always implicit in demands from smugglers: failure to comply creates physical risk not just from violence but from the danger of the environment.

### Formal and informal perpetrators and contexts of structural violence

The context of structural violence on the irregular migration journey intersects with economic need, a lack of agency, and the climate of violence overall that constructs a proximity to death. In terms of perpetrators, they can be formal and organised such as state actors, semi-formal and organised such as traffickers, and smugglers that are attached to gangs and organised crime. They can also be semi-formal without being organised, where a relationship of need creates a power disparity between a paid smuggler, or a guide, and this power disparity is exploited for sexual violence. Due to the extreme vulnerability constructed during the journey, opportune sexual violence also regularly occurs [62,66,72].

For migrants making irregular journeys, it makes little difference whether perpetrators are representatives of the state such as police or immigration officers, or whether they are organisations such as smugglers, traffickers, or organised criminal gangs. In both contexts the protective role is present but ambivalent (see above findings relating to ‘perpetrators’), and due to the nature of migration without state authorisation, there is a lack of pathway to accountability for violence for the perpetrators, or for the potential for protection from violence for the victims. While there are migrant shelters and safehouses along some of the routes travelled, these can only offer temporary protection. Routes for accountability are limited by lack of legal status in the transit country. Sexual violence often happens during the trajectory of the journey and the migrant victim-survivor continues to travel with the perpetrators. Alternatively it happens while navigating an immigration check and onward travel is gained via submission to sexual violence [52,57]. It is clear that at times there is collusion between state actors and smugglers and traffickers who are responsible for sexual violence [54,56,57]. In these contexts, the irregular nature of the route itself prevents the ability to report violence, to support future prevention of violence, or to seek accountability for violence on the part of the migrant.

There is additionally evidence of systematization of violence in the type and context of abuse. This includes group or collective sexual violence and forced witnessing. Violence is systematised in repeated patterns. There was evidence both that sexual violence can be randomised with groups forced to witness repeated violence creating a situation of terror, and sexual violence can also be associated with punishment for transgression or resistance [87]. Technology-enabled violence was also used in a systematised way to enforce long-term compliance among victims, recording torture and sexual violence to post on social media or threaten with public shame [51]. In these cases, sexual violence is employed as a tool of control and for financial gain. The presence of these patterns in the evidence suggests entrenched and embedded sexual violence in an immigration system that is passively permissive of it, and even benefitting from it in support of efforts to deter undocumented migration.

### Normalisation of violence: Gender dimensions

It was well-evidenced, and cut across the various interpretive codes, that violence against women was normalised and expected on the journey. There was very limited data on non-binary border crossers [68], and few studies dedicated specifically to sexual violence against men [58,78,87]. While sexual violence on the journey potentially affects all migrants, the gendered power dynamics shape these experiences [10]. Smugglers, traffickers, and border guards are generally male, meaning women are caught in a power dynamic whereby they rely on men to facilitate border crossing [56]. Sexual violence was discussed in terms that highlighted the inevitability and acceptance of the risk of sexual violence [5,18,10,72]. The most brutal forms of sexual violence, particularly torture and imprisonment over time, were an exception to the normalised language. While women expected to be at risk of sexual violence during the journey, they set out expecting to complete the journey. While this was the case for interviewee victim-survivors, there was a lot of evidence of torture, violence and deaths of women who either did not complete their journey, or there was no reason to believe they completed the journey. This type of sexual violence was widespread on the North African routes [19,34,56], but also discussed in relation to criminal gangs in Central America and Mexico [62].

Where violence is expected it is reported in a way that suggests it is experientially less severe than when it is unexpected. Of course, this is a value judgment guided by perception and may differ significantly by experience. However, considering how the normalisation of violence intersects with agency and the types of (constrained) empowerment adopted by women offers some insight. The most significant finding in this area was the intersection between normalised violence and gender-based violence. Violence against women was widespread, common, and often expected. Sexual violence against men was not as widespread as sexual violence against women, but was still common. This was particularly clear on the North African routes, and evidenced in Libya, but mentioned elsewhere [58,87]. Studies that reported sexual violence against men were initiated via health data, finding physical evidence of sexual violence that launched further qualitative investigation. Violence against women on migration journeys tends to be construed as an expected risk of irregular migration, violence against men remains hidden, associated with shame, and when discussed highlights the extreme brutality. While this reflects gendered power dynamics and female victimhood *in general*, it also suggests a tactic of violence that emasculates male migrants travelling without authorization in order to control, extort, and victimise them.

## Limitations of study

This study traced occurrences of sexual violence along migration routes. While the search was extensive and no studies were excluded on the basis of language, the search was performed in English, meaning the results biased towards English language research. This may underrepresent research produced in regions where migration flows and related violence are intense, but where publication typically occurs in other languages. The findings are also limited by the reality of the bias towards Global North resources in academic studies: the evidence included here represents the most frequently studied routes, but this does not necessarily mean that they are also the only routes along which sexual violence occurs. The routes transiting through Mexico and through Libya share the characteristic of travel from Global South to Global North states. Immigration deterrence in the Global North, specifically Europe and the USA, means that these routes are highly publicised in media and public discourse, with significant research resources deployed to better understand migration along them. There is little information available in this review about sexual violence on South-South transit routes. No East Asian routes were described in the included studies. This may be a consequence of the English language search. It may be a consequence of the ‘exposure’ criteria during transit, reflecting a particular structure of journey. It suggests a need futher research to identify under what conditions sexual violence occurs on these migration routes.

Finally, the review reflects the limitations of the underlying evidence base. Included studies varied in methodological rigour, sample size, and depth of reporting, which may influence how experiences of sexual violence were captured or interpreted. It is also possible that experiences of sexual violence may be underreported due to stigma, fear, or ethical constraints on data collection.

## Recommendations for future research

This study recommends that further research is carried out in mapping South-South routes of international migration to better understand the risks of violence associated with irregular movement globally. There is also a gap in understandings of potential accountability for sexual violence along migration routes. It is necessary to examine the support systems available to people travelling without documents, and where there are significant gaps in support that give rise to violence.

This research found that there are spatial and temporal aspects to sexual violence along migration routes. It is necessary to examine further how this type of violence fits into existing knowledge of intersecting forms of vulnerability to violence, and to understand the extent to which these vulnerabilities are replicated by producing likelihood of taking a migration journey and likelihood of experiencing violence during the course of such a journey. It is also useful to better understand the longer-term social implications of violence during migration journeys to know how people who have been victims of such violence can be supported in their communities of resettlement.

Finally, there is a lack of transparent information on how states respond to sexual violence along international migration routes. While there is clear and robust evidence that such violence takes place, there is little to suggest that states take effective preventative or punitive measures to tackle such violence. This may be due to lack of government capacity, lack of resources, or lack of political will, but the extent and scale of the violence in evidence suggests that governments should prioritise capacity and resources as a matter of urgency. This systematic review and analysis suggests that additional investigation to assess state action and outcomes is needed. The extent of the violence, and the lack of efforts to prevent violence towards irregular migrants, coupled with the rhetoric of immigration deterrence adopted by migrant-receiving states, suggests that states may welcome and appropriate violence as a means of immigration deterrence. This calls for further investigation.

## Conclusion

This study found extensive sexual violence along the most studied migration routes and along less studied migration routes. It did not cover all international irregular migration, but the evidence reviewed finds that sexual violence is certainly present, and is one of a set of violent tactics used to control, extort and victimise people who have no or very limited recourse for support. State governments do little to protect migrant victims of sexual violence, or migrant victims of violence in general [3,22,96,97]. Indeed, there is clear evidence to substantiate the claim that states use violence against migrants [98–101] and that states appropriate forms of social and domestic violence as part of the arsenal of immigration deterrence [96]. In this case, violence happens against migrants travelling without documentation. Such violence is robustly evidenced.

Available protection tends to be provided by the non-profit sector and from religious institutions rather than state services [57]. States have at times taken action to block funding from reaching such organisations because of the support they supply to undocumented migrants [102]. While brutal violence may occur on migration journeys, there is no mechanism available to prevent return to locations along the journey that are generically classed as ‘safe.’ These so-called safe destinations are often locations where violence victimisation has occurred and remains a significant threat [51,63].

Sexual violence emerges from this review as a structurally embedded feature of irregular migration. Economic precarity plays a role by increasing migrants’ vulnerability and constraining their choices in ways that increase exposure to sexual violence and coercion. In several studies, migrants demonstrated awareness of the risk of sexual violence and took measures to mitigate them, such as using contraception to prevent unwanted pregnancy or travelling with male companions to reduce their vulnerability. Many journeys take place in isolated environments, where smugglers and traffickers may be the only available means of passage, and refusing sexual contact may mean abandonment or death. The most severe forms of sexual violence are ruthless, indiscriminate, involve forced witnessing, and forced sex act perpetration on other migrants.

Although sexual violence affects migrants of all genders, there was variation by gender in experiences and expectations of violence across studies. Perpetrators of the violence included organised and systematic perpetration by state agents such as border guards and police, by smuggling and trafficking gangs, and opportunists such as fellow travelling migrants or people who reside along the route through which migrant populations transit. Sexual violence is structurally embedded in international migration systems and this demands an urgent response.

## Supporting information

S1 Table PRISMA Checklist. (DOCX)

S2 Table Search Terms. (DOCX)

S3 Table Exclusion chart. (DOCX)

S4 Table Quality Assessment. (DOCX)
