# Supplementary material for: Sexual violence along migration routes: A qualitative systematic review and evidence synthesis
Source: PLoS One. 2026 Aug 13;21(8):e0355474. doi: 10.1371/journal.pone.0355474 (PMC13472432; doi:10.1371/journal.pone.0355474)
Supplement: Supplementary file 2 [file pone.0355474.s002.docx]

## PEO Exclusion Table

|  | **Include** | **Exclude** |
| --- | --- | --- |
| **Report Type** | Peer reviewed academic journal articles  Reports  Dissertations | Book reviews  Conference papers  Systematic reviews (but use for additional source articles)  Magazine articles  Editorials |
|  |  | **Reason: Wrong report type** |
| **Study Type** | Qualitative empirical research  Mixed methods reporting qualitative findings | Theoretical or conceptual development with no primary data  Studies that are quantitative only  Studies that refer to data pre-2010. |
|  |  | **Reason: Wrong study type** |
| **Population** | People in undocumented migration status, or without any status, or with status pending who are not in their final intended destination of immigration.  People who are in transit from their home towards a country of immigration. | People who have completed immigration, people who have not yet left their home country, people who have received a ‘failed’ immigration determination, people who retain a valid immigration status for the duration of a journey. People who have status pending and are in their final intended destination of immigration.* |
|  |  | **Reason: Wrong population type** |
| **Outcome** | Sexual violence, including rape, sexual assault, sexual harassment, indecent exposure, forced penetration, forced prostitution, sexual favours by coercion (such as in exchange for food or shelter), coercive control through threat of sexual violence, including exposure and use of indecent images. | Violence that is not sexual in nature (such as assault, robbery, or other forms of aggression or threat). |
|  |  | **Reason: Wrong outcome** |
| **Exposure** | Sexual violence while undertaking a migration journey.  The journey must be underway when violence is experienced, and not yet complete.  The experiences of violence are recounted first hand by the population and are linked to their undocumented migration status.  The experiences of violence are recounted second hand by support workers, practitioners, or other professionals with regular contact with people in insecure migration status, and are linked to insecure migration status. | Violence that happened before the journey began or violence that caused migration (such as war, conflict, persecution, gang violence).  Violence that happened after immigration is complete in a destination country.  Violence that happened after an application for immigration has been filed. |
|  |  | **Reason: Wrong exposure** |
| **Notes**  *If intended destination is not stated, or intent to remain is unclear, include when immigration status is pending. | | |
