# Supplementary material for: Sexual violence along migration routes: A qualitative systematic review and evidence synthesis
Source: PLoS One. 2026 Aug 13;21(8):e0355474. doi: 10.1371/journal.pone.0355474 (PMC13472432; doi:10.1371/journal.pone.0355474)
Supplement: Supplementary file 3 [file pone.0355474.s003.docx]

**Sexual violence along migration routes**

**Concept clusters for search**

| **Sexual violence** | **Migration** | **Methods** |
| --- | --- | --- |
| “Forced sex*” | Asylum-seeker* | “Case stud*” |
| “Indecent assault” | “Asylum seeker*” | Empirical |
| “Indecent exposure” | Deport* | Ethnograph* |
| “Non-Consensual Intercourse | “Forced migra*” | Experiential |
| “Non-consensual sex*” | “Human smuggl*” | “focus group*” |
| Rape | “Human traffick*” | Interview* |
| “Sexual abuse” | “Immigration detention” | Participatory |
| “Sexual assault” | “Irregular migra*” | “Participant observation” |
| “Sexual exploitation” | Migrant* | Qualitative |
| “Sexual harassment” | “Migrant camp*” |  |
| “Sexual misconduct” | “Migration detention” |  |
| “Sexual violence” | Refoule* |  |
| Stalking | Smuggl* |  |
|  | Traffick* |  |
|  | Undocumented |  |
|  | “Without status” |  |

| **Database** | **Date** | **Results Combined Search** |
| --- | --- | --- |
| EMBASE (Ovid) | 10-1-25  16-1-25 | 186  202 |
| Sociology Source Ultimate | 10-1-25  16-1-25 | 186  217 |
| Political Science Complete | 13-1-25  16-1-25 | 34  34 |
| Social Policy and Practice | 13-1-25  16-1-25 | 75  75 |
| Web of Science SS | 13-1-25  16-1-25 | 269  269 |
| **TOTAL** | | **750**  **793** |
| **Duplicates** | | **258** |
| **Total abstracts to screen** | | **535** |

**Searches to paste into grey lit databases**

**Original:**

(“Forced sex*” OR “Indecent assault” OR “Indecent exposure” OR “Non-Consensual Intercourse” OR “Non-consensual sex*” OR Rape OR “Sexual abuse” OR “Sexual assault” OR “Sexual exploitation” OR “Sexual harassment” OR “Sexual misconduct” OR “Sexual violence” OR Stalking)

AND

(Asylum-seeker* OR “Asylum seeker*” OR Deport* OR “Forced migra*” OR “Human smuggl*” OR “Human traffick*” OR “Immigration detention” OR “Irregular migra*” OR Migrant* OR “Migrant camp*” OR “Migration detention” OR Refoule* OR Smuggl* OR Traffick* OR Undocumented OR “Without status”)

AND

(“Case stud*” OR Empirical OR Ethnograph* OR Experiential OR “focus group*” OR Interview* OR Participatory OR “Participant observation” OR Qualitative)

**Modified for OAIster:**

(“Forced sex*” OR “Non-consensual sex*” OR Rape OR “Sexual abuse” OR “Sexual assault” OR “Sexual exploitation” OR “Sexual harassment” OR “Sexual violence”)

AND

(“Asylum seeker*” OR Deport* OR “Forced migra*” OR “Human smuggl*” OR “Human traffick*” OR “Immigration detention” OR “Irregular migra*” OR Migrant* OR “Migrant camp*” OR “Migration detention” OR Refoule* OR Undocumented OR “Without status”)

AND

(“Case stud*” OR Empirical OR Ethnograph* OR Experiential OR Interview* OR Participa* OR Qualitative)

*Removed words*

- “Indecent assault”
- “Indecent exposure”
- “non-consensual intercourse”
- “Sexual misconduct”
- Stalking
- Asylum-seeker*
- Smuggl* OR Traffick*
- “Participant observation”
- “focus group*”
