# Supplementary material for: Sexual violence along migration routes: A qualitative systematic review and evidence synthesis
Source: PLoS One. 2026 Aug 13;21(8):e0355474. doi: 10.1371/journal.pone.0355474 (PMC13472432; doi:10.1371/journal.pone.0355474)
Supplement: Supplementary file 4 [file pone.0355474.s004.docx]

| **Appendix 3: Risk of bias quality assessment of fully included studies**  *Adapted from the Critical Appraisal Skills Programme (CASP) Checklist for Qualitative Research* | | | | | | | | | | |
| --- | --- | --- | --- | --- | --- | --- | --- | --- | --- | --- |
| **Study** | **Was there a clear statement of aims?** | **Is a qualitative method appropriate?** | **Was the research design appropriate to address aims?** | **Was the recruitment strategy appropriate to address aims?** | **Was data collected in a way that addressed the research issue?** | **Has the relationship between researcher and participant been considered?** | **Were ethical issues considered?** | **Was analysis sufficiently rigorous?** | **Is there a clear statement of findings?** | **Is the research valuable?** |
| Adeyinka et al. 2023 | ** | ** | ** | * | ** | ** | ** | ** | ** | ** |
| Brigden 2018 | * | ** | ** | ‡ | * | ** | - | ‡ | * | ** |
| Cenat et al. 2020 | ** | ** | ** | ** | ** | - | - | ** | ** | ** |
| Chynoweth et al. 2022 | ** | ** | ** | ** | ** | - | ** | ** | ** | ** |
| Cook Heffron 2019 | ** | ** | ** | ** | ** | ** | ** | ** | ** | ** |
| Cortés 2018 | ** | ** | * | - | * | - | - | - | ** | ** |
| Elmagboul et al. 2017 | ** | ** | ** | * | * | - | - | * | ** | ** |
| Gebreyesus et al. 2019 | ** | ** | ** | ** | ** | - | ** | ** | ** | ** |
| Ghebrezghiabher and Motzafi-Haller 2015 | ** | ** | ** | ** | - | ** | - | - | ** | ** |
| Goodson et al. 2020 | ** | ** | ** | - | * | - | - | - | ** | * |
| Infante et al. 2013 | ** | ** | ** | ** | ** | - | * | ** | ** | ** |
| Infante et al. 2020 | ** | ** | ** | ** | ** | - | ** | ** | ** | ** |
| Izcara Palacios and Andrade Rubio 2022 | ** | ** | ** | * | ** | - | ** | - | ** | ** |
| Keygnaert et al. 2014 | ** | ** | ** | ** | ** | ** | ** | ** | ** | ** |
| KIND and CDH Fray Matías 2017 | ** | ** | ** | * | ** | - | - | - | ** | ** |
| Letona et al. 2023 | ** | ** | ** | ** | ** | - | ** | ** | ** | ** |
| Leyva-Flores et al. 2019 | ** | ** | ** | ** | ** | - | ** | ** | ** | ** |
| López-Domene et al. 2019 | ** | ** | ** | ** | ** | - | ** | ** | ** | ** |
| Matose et al. 2022 | ** | ** | ** | ** | ** | ** | * | ** | ** | ** |
| Obach et al. 2022 | ** | ** | ** | ** | ** | - | ** | ** | ** | ** |
| Pertek et al. 2021 | ** | ** | ** | ** | ** | * | ** | * | ** | ** |
| Preko 2022 | ** | ** | ** | * | * | - | - | * | ** | ** |
| Rubini et al. 2024 | ** | ** | ** | ** | ** | N/A | ** | ** | ** | ** |
| Soria-Escalante et al. 2022 | ** | ** | ** | ** | ** | * | ** | ** | ** | ** |
| UNICEF 2016 | ** | ** | * | - | * | - | - | * | ** | ** |
| Vogt 2012 | ** | ** | ** | - | * | ** | ** | * | ** | ** |
| WRC 2019 | ** | ** | ** | ** | ** | - | ** | * | ** | ** |

**criterion completely satisfied; *criterion partially satisfied; - unclear or insufficient information; ‡criterion not satisfied; N/A Not applicable
